# Severe Fetal Anaemia Secondary to Fetomaternal Haemorrhage: A Case Report Exploring Aetiologies and Diagnostics

**DOI:** 10.7759/cureus.105632

**Published:** 2026-03-21

**Authors:** Aaron S Lawrence, Ajith Samaratunga

**Affiliations:** 1 Obstetrics and Gynaecology, Dubbo Hospital, Dubbo, AUS; 2 Obstetrics and Gynaecology, Royal Prince Alfred Hospital, Sydney, AUS

**Keywords:** advanced maternal age, fetal anaemia, fetomaternal haemorrhage, placental histopathology, thrombocytopenia in pregnancy

## Abstract

Clinically significant fetomaternal haemorrhage (FMH) is a rare but significant cause of fetal anaemia, often associated with placental or umbilical cord abnormalities and with obstetric manoeuvres and traumatic events. This case follows a 40-year-old woman in her second pregnancy who sustained a severe, unprovoked FMH resulting in an emergency caesarean section at 34+3 weeks' gestation. While her newborn required cardiopulmonary resuscitation and birth and subsequent respiratory support (including an admission to the neonatal intensive care unit), there was a good recovery. This patient's case is of interest due to the lack of major risk factors (including a reassuring fetal growth and well-being scan just two days prior) and raises questions about screening, risk stratification and effective diagnosis. These can assist in identifying women at the highest risk of developing FMH and facilitate rapid diagnosis in order to minimise maternal and neonatal morbidity and mortality.

## Introduction

Fetomaternal haemorrhage (FMH) is an important cause of fetal anaemia and describes the passage of fetal blood into the maternal circulation across the placental interface. It is thought that a degree of FMH occurs in all pregnancies, likely related to the thinning of the placental barrier as a pregnancy progresses [1-3]. Clinically significant FMH is rare (estimated to be three pregnancies for every 1000) and refers to the passage of more than 30 mL of fetal blood into the maternal circulation [2].

There are a number of conditions and triggering events that have known associations with FMH, including pre-eclampsia, placenta praevia spectrum disorders, placenta accreta spectrum, cord-related accidents (including vasa praevia, cord prolapse, cord avulsion, nuchal cord) and placental tumours (including chorioangiomata). Further, trauma (motor vehicle accidents primarily) is also associated with FMH, as are manoeuvres such as external cephalic version, manual removal of placenta, amniocentesis and chorionic villous sampling [2-5]. However, these triggers account for at most 20% of FMHs [1,2].

The most common symptom reported in presentations with FMH is decreased fetal movements; however, it is non-specific and can be seen even in benign presentations [2,6,7]. Cardiotocography (CTG) should be performed in all such presentations; a sinusoidal CTG is the classic sign indicating clinically significant fetal anaemia that may require urgent delivery. This is not sensitive, but is highly specific, and the CTG pattern is most closely associated with FMH [8].

The definitive diagnosis of FMH is based on laboratory testing; the Kleihauer-Betke test has long been the standard. This relied on manually counting the number of fetal haemoglobin (HbF)-containing cells able to withstand acid elution on a peripheral blood smear; however, this has largely been superseded by flow cytometry-based approaches assessing HbF and carbonic anhydrase levels [9-11].

Management takes one of two paths: either stabilising and resuscitating the fetus or delivering emergently [3,12,13]. This decision is guided by fetal welfare and is a balance between prematurity-related morbidity and the morbidity sustained by the FMH.

This case follows a 40-year-old woman in her second pregnancy who presented at 34+3 weeks' gestation with absent fetal movements for 4-6 hours on a background of reduced fetal movements over the preceding 24 hours.

## Case presentation

**Figure 1:**
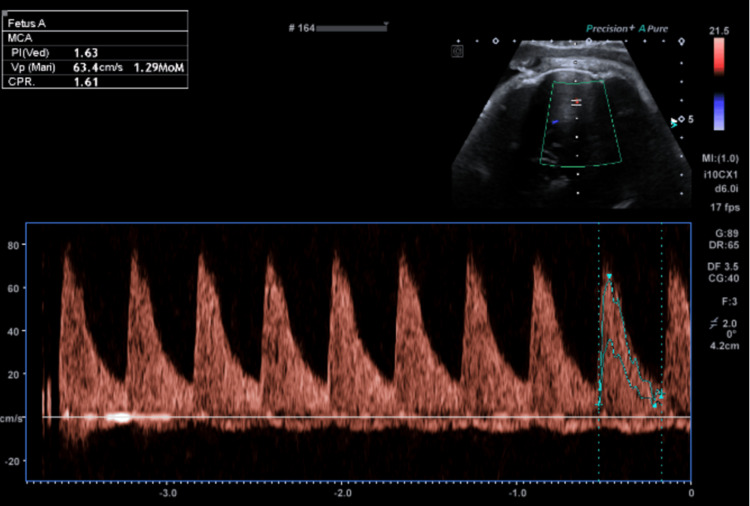
Image from the fetal growth and well-being scan at 34+1 weeks' gestation during the assessment of cranial blood flow, demonstrating the MCA, CPR and MCA-PSV (all fall within normal range). MCA: middle cerebral artery; CPR: cerebroplacental ratio; MCA-PSV: middle cerebral artery peak systolic velocity

**Figure 2:**
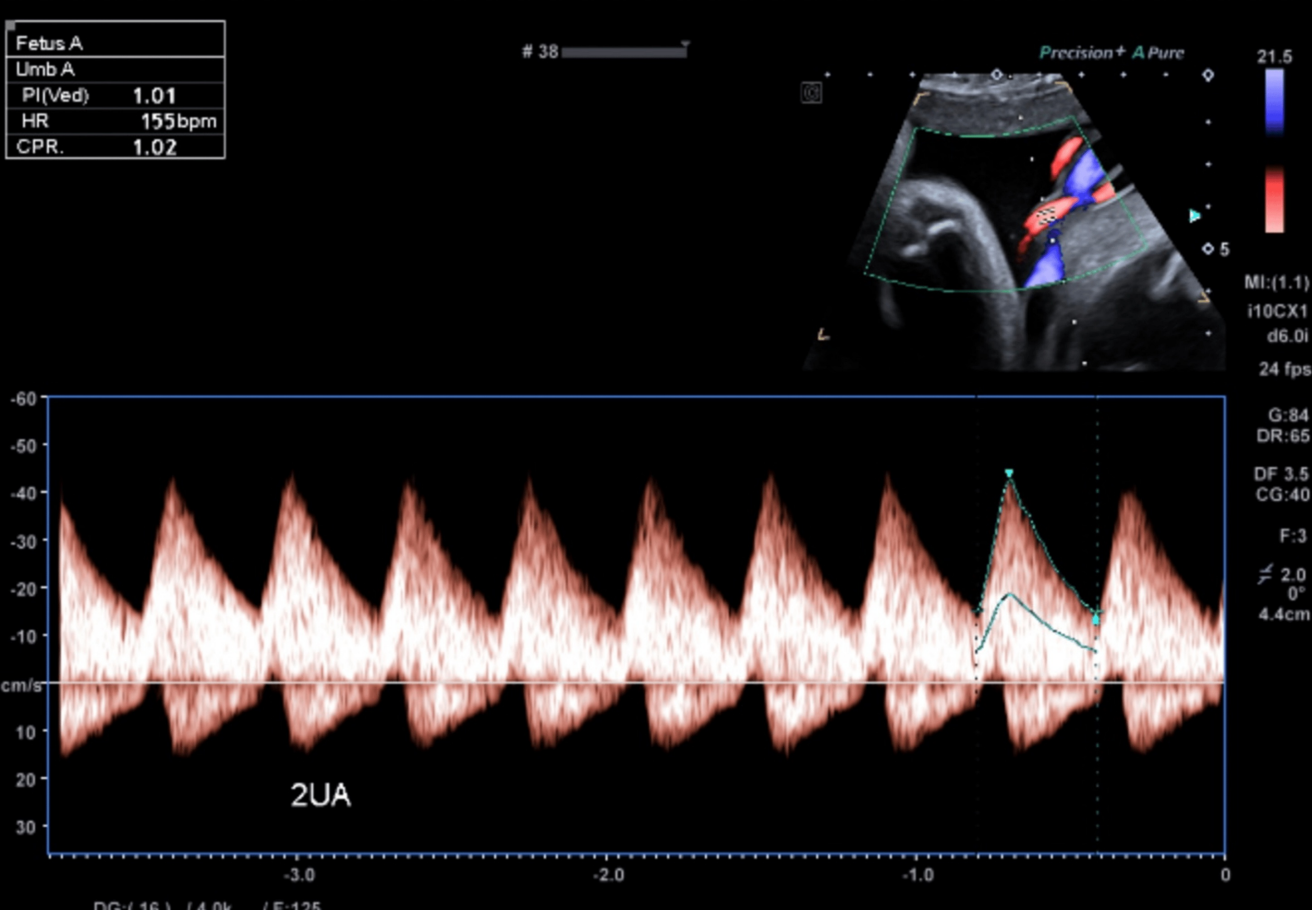
Image from the fetal growth and well-being scan at 34+1 weeks' gestation during the assessment of UAPI and CPR (both within normal range). UAPI: umbilical artery pulsatility index; CPR: cerebroplacental ratio

This case follows a 40-year-old woman in her second pregnancy, conceived by assisted reproductive technology and complicated by thrombocytopenia (gestational favoured, platelet count 116×10^9^/L on the day of presentation). This pregnancy was otherwise low risk, and she was cared for by the midwifery clinic in her local hospital catchment. She had no significant medical history, was of an O-positive blood group with no known antibodies, and had been on low-dose aspirin during the pregnancy on account of her age and having conceived by in vitro fertilisation (IVF). No issues had been noted on her morphology scan nor on a growth and well-being scan at 34+1 weeks' gestation (performed due to risks conferred by IVF, advanced maternal age and thrombocytopenia). Dopplers at this recent ultrasound included a middle cerebral artery pulsatility index (MCAPI) of 1.63 (Figure 1), an umbilical artery pulsatility index (UAPI) of 1.01 (Figure 2) and a cerebroplacental ratio (CPR) of 1.61 (Figure 1). The middle cerebral artery peak systolic velocity (MCA-PSV) was 63.4 cm/s (Figure 1). Of note, this patient's previous pregnancy was also complicated by thrombocytopenia and culminated in a vacuum-assisted delivery without any complications.

**Figure 3:**
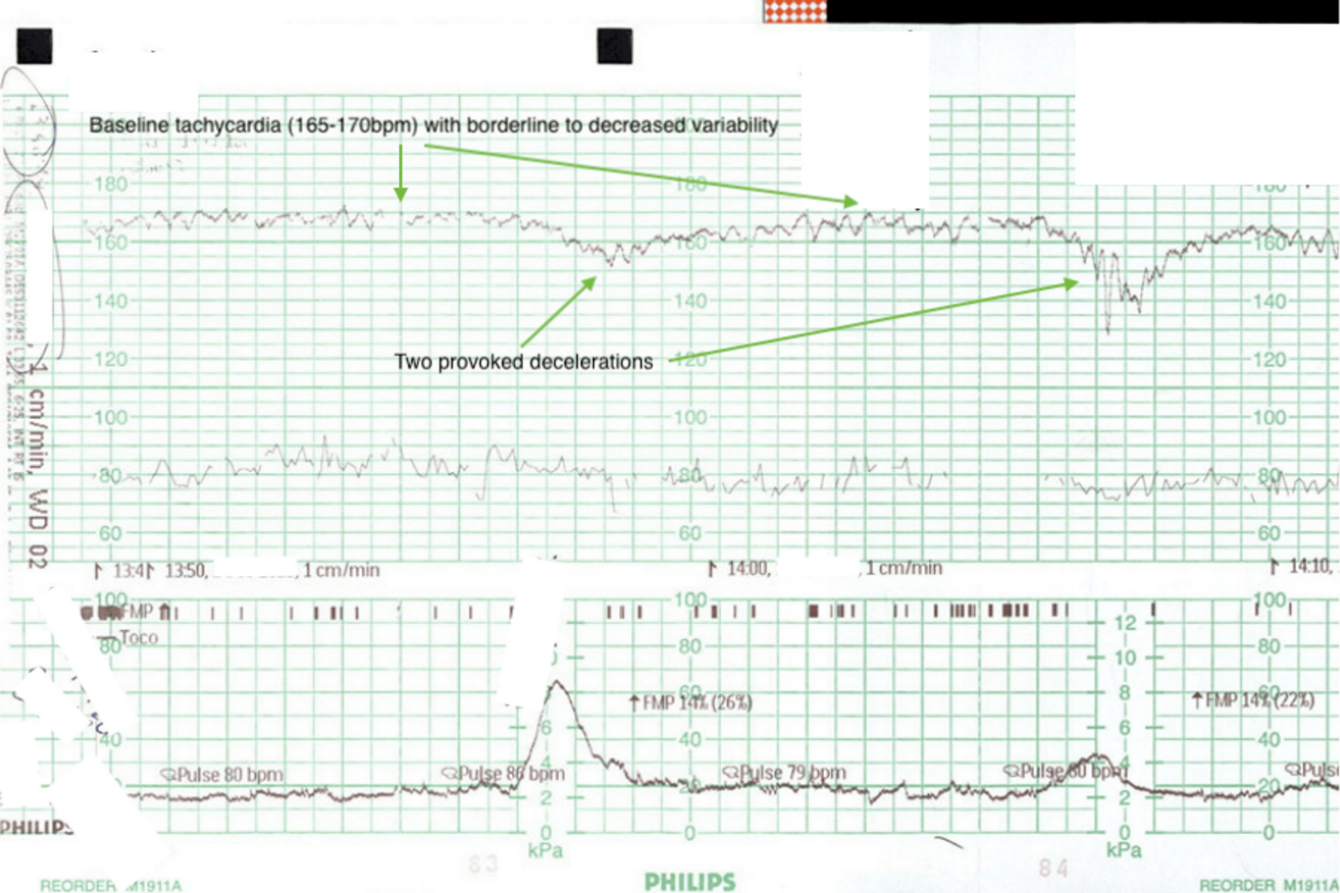
First part of the CTG commenced on arrival to the Day Assessment Unit. Can note the baseline tachycardia and two provoked decelerations: the first shallow and the second deeper. Variability is also borderline. CTG: cardiotocography

**Figure 4:**
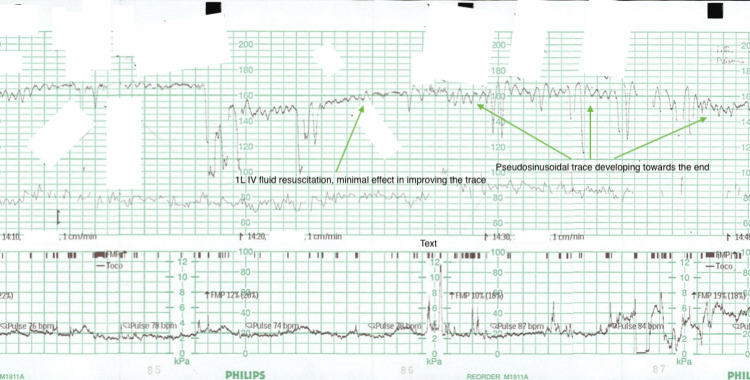
Second part of the CTG; still retains an elevated baseline and borderline variability. Minimal improvement with the administration of 1 L intravenous fluids at the marked time point. Trace starts to become pseudosinusoidal, and a decision for a caesarean section is made at the end of this snippet. CTG: cardiotocography

At 34+3 weeks' gestation, this patient presented to the Day Assessment Unit, a walk-in service provided by the obstetrics and gynaecology department in her local rural centre. She described absent fetal movements for 4-6 hours on a background of reduced fetal movements over the preceding 24 hours. CTG was promptly attended, demonstrating a baseline tachycardia (165-170 bpm), absent accelerations, decreased variability and provoked decelerations (illustrated in Figure 3 below). She was resuscitated with intravenous fluids, but this was unsuccessful in improving the CTG trace (illustrated in Figure 4 below). Shortly afterwards, the trace began to demonstrate pseudosinusoidal changes, and a category A caesarean delivery under general anaesthetic was promptly arranged due to concerns for fetal distress.

An uncomplicated caesarean section was performed within 30 minutes. Clear liquor was noted, and no retroplacental clot or cord factors were identified. A female infant was promptly delivered floppy, pale and without respiratory effort. She was taken immediately to be resuscitated in the anaesthetics bay by the paediatric team. Cardiopulmonary resuscitation was performed for four minutes, and she was then transitioned to intermittent positive pressure ventilation (IPPV) for a further five minutes and 40 seconds. At nine minutes and 40 seconds of life, she was transitioned onto continuous positive airway pressure (CPAP), and urgent transfer via fixed-wing air ambulance was arranged to a tertiary centre near the health district with a neonatal intensive care facility to provide further respiratory support. Her Apgars were 2 at one minute of life, 3 at five minutes and 7 at 10 minutes.

Initial blood tests taken from the patient's baby at the time of delivery demonstrated a haemoglobin of just 20 g/L (normal range 120-225 g/L, platelet count fibrin only, likely in the context of consumption). Prior to her transfer, she received two transfusions; the first took her haemoglobin to 57 g/L (platelet 135×10^9^/L), and after the second, her haemoglobin was 100 g/L. An FMH quantitation test taken at the time of initial presentation eventually returned a result demonstrating a 134 mL FMH (flow cytometry-based).

After a period of care in neonatal intensive care and special care, the patient's baby recovered with a right-sided grade I intraventricular haemorrhage which had resolved by the time of her discharge home. The patient also remained well (haemoglobin 106 g/L and platelet 121×10^9^/L the morning after delivery), without any lasting impact on her health.

## Discussion

This case highlights questions about FMH and fetal anaemia, including possible aetiologies in the absence of clear risk factors, and new investigations that can assist in facilitating timely intervention that optimises outcomes.

While this case was eventually confirmed as an FMH, this was not apparent at the time of her baby's birth with a haemoglobin of 20 g/L. As such, key causes of fetal anaemia needed to be excluded. The most common causes are haemolytic disease of the newborn (typically secondary to maternal alloimmunisation), as well as bone marrow suppression secondary to viral infection (principally parvovirus B19, but cytomegalovirus has also been implicated) [1,6,7,14]. This patient did not demonstrate any rashes or viral prodrome that might indicate an infective cause for fetal anaemia, nor did she have any known circulating antibodies.

Of note, this patient had thrombocytopenia in pregnancy (as in her first pregnancy). Importantly, maternal immune thrombocytopenic purpura can be associated with fetal and neonatal alloimmune thrombocytopenia (FNAIT), which can increase the risk of sequelae from bleeding. This patient had a normal platelet count of 200×10^9^/L three months prior to conception (reflecting a return to normal range after her first pregnancy) and 160×10^9^/L in the mid-second trimester. Serial platelet counts in the third trimester had been steady between 124×10^9^/L and 128×10^9^/L, with the nadir being on the day of presentation. These are more consistent with criteria for a diagnosis of gestational thrombocytopenia, although a platelet count 6-8 weeks postpartum would be beneficial for completeness [15-17]. Moreover, the finding of fibrin only on the initial neonatal platelet count in this case is more likely reflective of consumption given a return to 135×10^9^/L after a transfusion [18]. It is also worth noting that Wiedmeier et al. suggested that this platelet count is not truly thrombocytopenic in neonates born at this gestation [19].

As discussed above, a number of conditions and triggering events have been found to be associated with FMH. However, the pathophysiology underlying FMH is still yet to be fully understood. The placenta is considered to be implicated. Pathological analysis of placentae from pregnancies affected by FMHs has noted the rupture of distal villi, intervillous thrombi, placental infarcts and retroplacental haematomata [20,21]. Nucleated red cells, often seen in the blood of fetuses affected by hypoxia, have also been found in the intervillous space of placentae involved in FMHs [20]. None of these were found on this patient's placenta, but rather a marginal cord insertion, a hypocoiled umbilical cord and two chorionic septal cysts.

Abnormal coiling of the cord (both hypocoiling and hypercoiling) are associated with intrauterine fetal death and fetal growth restriction. However, this is typically due to some vascular obstruction rather than FMH [22].

While a marginal cord insertion was noted in this patient's case, it is interesting that this was not noted on her ultrasound scans. Siargkas et al. [23] performed a meta-analysis on studies assessing outcomes from marginal cord insertions, and while they did not assess for FMH specifically, they found loose associations with key outcomes from this case (including emergency caesarean deliveries and one-minute and five-minute Apgar scores less than 7). Importantly, they found significant associations with preterm delivery, caesarean delivery overall and neonatal intensive care unit admission. Wada et al. [24] assessed a Japanese population for FMH specifically, finding a positive but non-significant association with abnormal cord insertions.

Interestingly, chorionic cysts (including chorionic septal cysts) have some associations with chronic (rather than acute) hypoxia [25]. Despite this, the patient in this case had an MCAPI of 1.63 (19%ile, measured in Figure 1), a UAPI of 1.01 (70%ile, measured in Figure 2), and a CPR of 1.61 (15%ile, also measured in Figure 1) on her scan just two days prior to presenting. All of these are within normal limits, which would not be expected with hypoxaemia [26]. This may indicate the need for other ultrasonographic measures of placental function in patients who may be considered high risk for placental pathology. This patient may have been at increased risk due to being of advanced maternal age and having conceived by IVF.

Indeed, Torous and Roberts [27] surveyed the literature on placentae in pregnancies of advanced maternal age, comparing them to controls and finding some loose associations with fetal vascular malperfusion, delayed villous maturation and chorangiosis. (It is worth noting though that methodologically, the control group would typically include placentae sent for pathology only because the mother had some known risk factor such as pre-eclampsia or gestational diabetes.) Further, Kong et al. [28] and Siargkas et al. [29] have both studied the links between IVF and placental dysfunction. There are associations with placental conditions known to trigger FMH (placenta praevia, vasa praevia, placental abruption and so on), but no clear association with decreased placental function beyond this [28,29].

The difficulty in identifying clear factors that explain the majority of FMH cases suggests a need for further research in this area and also increases the importance of accurate and timely diagnostic techniques for FMH.

As discussed, the most common symptom associated with FMH is decreased fetal movements (trauma next most common), and a sinusoidal CTG (highly specific for severe fetal anaemia) is the most common trace associated with FMH [2,6,7]. A case series review by Bellussi et al. [8] identified two-thirds of FMH as having a sinusoidal CTG, whereas all cases had an abnormal CTG more generally including those demonstrating fetal tachycardia, non-reactive traces and decelerations. Figure 3 and Figure 4 demonstrate the abnormal trace noted in this patient's case.

Beyond these, the MCA-PSV is a highly sensitive test for severe fetal anaemia; Bellussi et al. noted that all but one of their reviewed cases had an MCA-PSV 1.5 multiples of the median (MoM), with 40% of their cases having an MCA-PSV greater than 2.0 MoM [8]. An MCA-PSV greater than 1.5 MoM is typically considered indicative of severe fetal anaemia; this is not specific to FMH though [13]. This patient's recent growth and well-being scan at 34+1 weeks' gestation demonstrated an MCA-PSV of 1.29 MoM (see Figure 1); despite histopathological findings, this suggests chronic anaemia secondary to FMH is less likely. Of note, MRI is more specific than MCA-PSV for fetal anaemia but is neither cost-effective nor practical in the urgent presentation [7].

While none of fetal movements, CTG and MCA-PSV are highly specific or sensitive for FMH on their own, the combination of the three could be useful as a form of "triple test" in the diagnosis of FMH. There is also the possibility of using these markers and other key risk factors in creating a risk score for FMH, potentially helping to stratify pregnancies; this data will likely need to come from long-term cohort studies given the paucity of research assessing correlations with FMH.

The Kleihauer-Betke test has long been the mainstay of FMH quantification; the traditional manual enumeration based on acid elution has been scrutinised as it is time-consuming and overestimates results since a small fraction of HbF is physiological in adult blood [9,10]. The more modern flow cytometry-based approach is more accurate in producing results but remains time-consuming and requires specially trained laboratory staff and expensive, specialised equipment [10,11]. While an accurate quantification is ultimately achieved, it does not allow a rapid indication of diagnosis in acute settings like in this case.

Peruzzi et al. [10] described a method using high-performance liquid chromatography to compare samples from normal adult blood against those suspicious for FMH on their haemoglobin composition. While this does not provide quantitation, it is possible to identify samples reflective of FMH after just eight minutes, providing further diagnostic clarity early in a patient's presentation.

Assunpção Nishio et al. [11] described the use of an electrical immunosensor based on the detection of cellular marker CD71 to identify the presence of fetal blood cells. Their method, based on measuring capacitance signal intensity and electrical impedance, is able to produce interpretable results with just a three-minute incubation time. This has potential as a point-of-care test that might drastically improve the efficiency with which a preliminary quantitation of FMH might be produced, again improving diagnostic clarity.

As already touched on, management decisions are dependent on gestation and fetal welfare and require multidisciplinary discussion between obstetricians and neonatologists. If the fetus is otherwise well, monitoring the FMH, administering steroids and considering an intrauterine transfusion are potential courses of management. Devlieger et al. suggested that intrauterine transfusions can be given as late as 34-35 weeks' gestation, allowing time for further maturation and development [3,12]. However, in the case of a pregnancy later than 34 weeks, delivery may be preferable [13]. Given the acuity of this presentation and clearly demonstrated fetal welfare, delaying would not have been an option, and urgent delivery was indicated. In such an acute context, this case also demonstrates the need for research into better diagnostic and risk stratification techniques; liquid chromatography, electrical impedance-based techniques and potentially the triple test proposed above may help bridge this gap.

## Conclusions

FMH is a clinically significant cause of fetal anaemia that is life-threatening yet often has few heralding signs and symptoms. This case, in a patient with an otherwise uncomplicated pregnancy in rural New South Wales, illustrates the importance of timely care in women with decreased fetal movements.
